# One fledgling or two in the endangered Carnaby's Cockatoo (*Calyptorhynchus latirostris*): a strategy for survival or legacy from a bygone era?

**DOI:** 10.1093/conphys/cou001

**Published:** 2014-02-17

**Authors:** Denis A. Saunders, Peter R. Mawson, Rick Dawson

**Affiliations:** 1 CSIRO Ecosystem Sciences, GPO Box 1700, Canberra, ACT 2601, Australia; 2 Perth Zoo, 20 Labouchere Road, South Perth, WA 6151, Australia; 3 Department of Parks and Wildlife, Locked Bag 104, Bentley DC, WA 6983, Australia

**Keywords:** *Calyptorhynchus latirostris*, Carnaby's Cockatoo, clutch size, fledging success, nestling health

## Abstract

Of the five species of black cockatoo in the genus *Calyptorhynchus*, those species with red tail bands (Red-tailed Black Cockatoo and Glossy Black Cockatoo) lay clutches of only one egg and those with white or yellow tail bands (Carnaby's Cockatoo, Baudin's Cockatoo and Yellow-tailed Black Cockatoo) usually lay clutches of two. The breeding of the endangered Carnaby's Cockatoo has been studied from 1969 to 2012 at a number of localities throughout its range in south-western Australia within a region largely cleared for agriculture. When raising nestlings the species feeds on seeds of native vegetation, and there was a strong but not significant negative relationship between nesting success and percentage loss of native vegetation within 6 and 12 km of nest hollows. There was a significant negative relationship between the health of nestlings and percentage loss of native vegetation around nest hollows. While the usual clutch size is two, average clutch size tended to be lower in areas where much native vegetation has been cleared. While both eggs hatch in 77% of two-egg clutches, the species normally fledges only one young. However, the species is capable of fledging both nestlings from a breeding attempt. Sets of siblings are usually the product of older, more experienced females nesting in areas where more native vegetation has been retained. The conservation implications of these findings are discussed in the light of predicted changes to the climate of south-western Australia.

## Abstract (precis)

Carnaby's Cockatoo is an endangered cockatoo endemic to Southwestern Australia. It normally lays two eggs with a median interval between them of eight days, but usually only fledges one nestling. Older, more experienced females are capable of fledging both young under conditions when food is not limiting. This can result in an increase in annual fledgling production. Predicted changes in climate of SW Australia may have major adverse consequences on breeding in the species.

## Introduction

In the Psittaciformes, despite the variation in the number of eggs in the clutches of some species, there is an inverse relationship between average clutch size and body weight (Saunders *et al.*, 1984). The Australian cockatoos (subfamily Cacatuinae) are the largest species of Australian parrots, and most members conform to this pattern [Gang Gang Cockatoo (*Callocephalon fimbriatum*), Palm Cockatoo (*Probosciger aterrimus*) and the ­members of the genus *Cacatua*; Galah (*C. roseicapilla*), Major Mitchell Cockatoo (*C. leadbeateri*), Little Corella (*C. sanguinea*), Long-billed Corella (*C. tenuirostris*), Western Corella (*C. ­pastinator*) and Sulphur-crested Cockatoo (*C. galerita*)].

The five recognized species of black cockatoo in the genus *Calyptorhynchus* [viz. Red-tailed Black Cockatoo (*C. ­banksii*), Glossy Black Cockatoo (*C. lathami*), Yellow-tailed Black Cockatoo (*C. funereus*), Baudin's Cockatoo *(C. baudinii*) and Carnaby's Cockatoo (*C. latirostris*)] do not conform to this pattern. Red-tailed Black Cockatoo (Saunders, 1977; body weight 530–870 g) and Glossy Black Cockatoo (Garnett *et al.*, 1999; 400–460 g) lay single-egg clutches, while clutch size is variable in Yellow-tailed Black Cockatoo (Saunders, 1979a; 505–900 g) and Carnaby's Cockatoo (Saunders, 1982; 480–790 g; weights and clutch sizes from Saunders, 2009), but clutches of two predominate. Little published information is available on the breeding of Baudin's Cockatoo (560–770 g). In Yellow-tailed Black Cockatoo and Carnaby's Cockatoo, when both eggs hatch, the younger nestling usually comes from a smaller egg and dies within 1–2 days of hatching (Saunders, 1982; Higgins, 1999; Forshaw, 2002).

**Figure 1:**
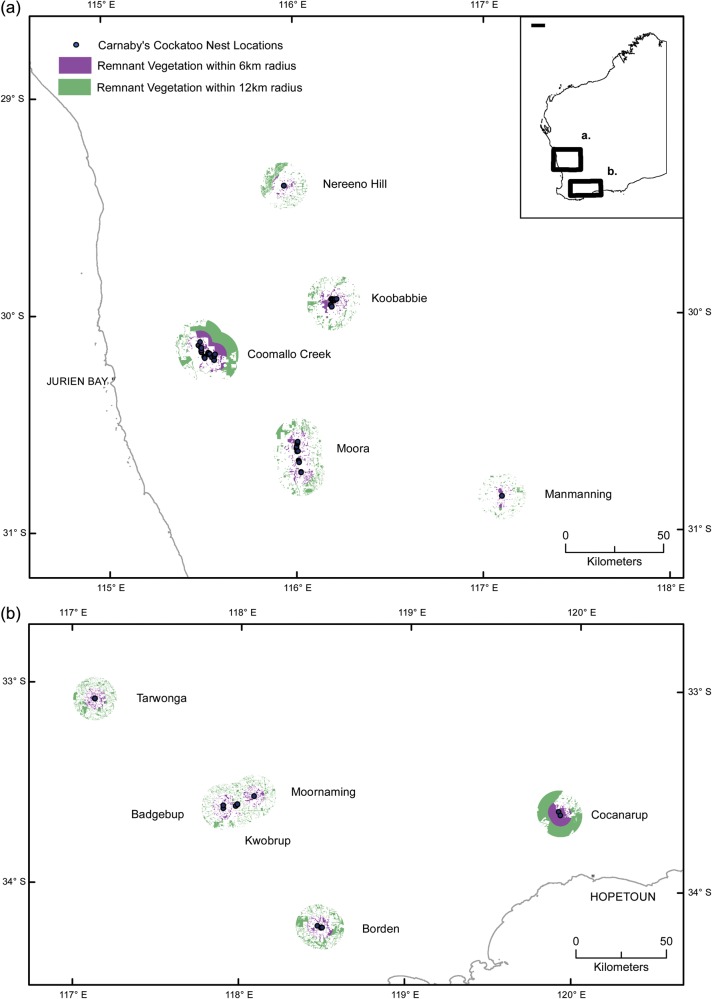
Location of each study area that produced two nestlings aged 14 days or more and the extent of native vegetation remaining within a 6 and 12 km radius of the nest hollows in the ‘Midlands’ (**a**) and ‘Great Southern’ (**b**).

Carnaby's Cockatoo is endemic to south-western Australia. It is listed as endangered under the Australian *Environment Protection and Biodiversity Conservation Act 1999* and as ‘Fauna that is rare or likely to become extinct’ in Schedule 1 of the Western Australian *Wildlife Conservation (Specially Protected Fauna) Notice 2012(2)* under the *Wildlife Conservation Act* 1950. The ecology and behaviour of this species have been studied in detail since 1969 (Saunders, 1982; Saunders and Ingram, 1998). Those studies were based on two populations; at Coomallo Creek and Manmanning, both in the northern wheatbelt. In addition, the species has been studied in less detail at Nereeno Hill and in the wider ‘Midlands’ region of the northern wheatbelt and at Tarwonga, Moornaming and the wider ‘Great Southern’ region in the southern wheatbelt (Fig. 1).

Based on studies at Coomallo Creek and Manmanning, Saunders (1982) reported that Carnaby's Cockatoo lays one or two eggs, with two being more common. Average clutch size was larger at Coomallo Creek than at Manmanning. Saunders also reported that in two-egg clutches, when both eggs hatched, the younger nestling most commonly died within 48 h of hatching. That study showed that between 1970 and 1976 at Coomallo Creek, eight of 222 (3.6%) nesting attempts fledged both nestlings. During the same period at Manmanning there were no instances of two nestlings from the same nest hollow surviving to fledge. A review of field data collected on Carnaby's Cockatoo since the study reported by Saunders (1982) at Coomallo Creek and in other areas has also shown that a number of hollows in which two eggs are laid result in two nestlings that survive for longer than 1–2 days; in some cases, both nestlings have fledged.

This successful fledging of both nestlings raises a number of questions, such as the following: in what proportion of nesting attempts does this occur; is the raising of two nestlings achieved at a cost to one or both nestlings; is there a net benefit to the species in being able to fledge both nestlings; and does the species' current environment support such a strategy?

In view of the conservation status of Carnaby's Cockatoo and the need for the best available information to guide conservation and management of this endangered species, we present data on clutch size from six locations, covering a ­considerable part of the range of the species during the period 1969–2012, with data recorded from 2 to 26 years at each location during 34 of the 44 years of the study period. We also present data on the clutch size of known individual females over time, the incidence of both eggs from one breeding attempt producing nestlings that survive for more than 14 days and the incidence of both nestlings from one breeding attempt successfully fledging. We provide data on individually marked females known to have fledged both nestlings successfully and information on individually marked siblings seen after fledging. We provide data indicating the health of nestlings, including siblings, while in the nest hollow that may foreshadow their likelihood of surviving after fledging. We also provide data on the relative health of nestlings from 10 locations relative to the amount of native vegetation cleared within a 6 and 12 km radius of each location. The conservation implications of this breeding behaviour are discussed in the light of predicted changes in the climate of south-western Australia.

## Materials and methods

### Study areas

Between 1969 and 2012, the breeding of Carnaby's Cockatoo was studied at 10 locations throughout much of the species' breeding range (Fig. 1). In each of these locations, Carnaby's Cockatoo nested in hollows in large eucalypt trees and when raising nestlings fed on seeds of native plants, mainly of the family Proteaceae, along with insect larvae which parasitize native plants (Saunders, 1980). All of the study locations were in the wheatbelt, a region that has been extensively cleared of native vegetation to facilitate broad-scale agriculture, mainly the production of cereal crops and grazing of domestic livestock on pastures of non-native plant species (Jarvis, 1981).

The most detailed studies over the longest period (1969–2012) were conducted at Coomallo Creek (30° 08′S; 115° 30′E; 35% remnant vegetation remaining within a 6 km radius of the study site). The population in this area was monitored from once to 25 times from July through to January during the breeding season for 26 of the 44 years (the number of visits to each study location is shown in Supplementary Table 1). In the early part of the study until the mid-1970s, only 10% of the study area had been cleared of native vegetation. By the 1980s more than 50% of the area had been cleared of native vegetation, and by the mid-1990s 65% had been cleared. A complete description of the Coomallo Creek area and changes in the extent of native vegetation in the area from 1969 to 1996 are given by Saunders and Ingram (1998). The Manmanning area (30° 51′S; 117° 06′E; 9% remnant vegetation), described by Saunders (1980), was visited from seven to 21 times each season from 1969 to 1976. The Carnaby's Cockatoo population was extirpated from the area in 1977 as part of a widespread decline in range as a result of loss of habitat due to clearing of native vegetation for agricultural development (Saunders, 1990). Tarwonga (33° 05′S; 117° 08′E; 20% remnant vegetation) was visited between two and 10 times each year from 1969 to 1971. Moornaming (33° 34′S; 118° 05′E; 16% remnant vegetation) was visited three times in 1969 and seven times in 1970. Nereeno Hill (29° 25′S; 115° 57′E; 9% remnant vegetation) was visited between two and 11 times each year between 1975 and 1980. Carnaby's Cockatoo nest hollows in the ‘Midlands’ [Moora (11% remnant vegetation) and Koobabbie (16% remnant vegetation)], which extends from Coorow in the north, south almost to Chittering (30° 02′S; 115° 31′E to 31° 15′S; 116° 47′E), were visited from three to nine times each year between 1996 and 1998 and twice each year during 2003–2012 (with the exception of 2004, when no field work was conducted). Two specific sites, Koobabbie and Moora (Fig. 1), were studied in more detail with regard to the health of nestlings relative to surrounding habitat quantity. Nests in the ‘Great Southern’ (Fig. 1), which extended from east of Katanning to Dragon Rocks and south to the Stirling Range (33° 36′S; 117° 58′E to 34° 13′S; 119° 54′E), were visited only once each season during 2008–2012. Three specific sites, at Borden (17% remnant vegetation), Kwobrup and Badgebup Nature Reserves (13% remnant vegetation) and Cocanarup Timber Reserve (75% remnant vegetation), were studied in more detail with regard to the health of nestlings relative to surrounding habitat quantity.

**Table 1:** Clutch size, hatching and fledging success for Carnaby's Cockatoo at six study locations

Location	Year	No. of nests	No. with one egg	No. hatched (%)	No. fledged (%)	No. with two eggs	No. hatched (%)	No. fledged (%)	Clutch size (mean ± SD)
Coomallo Creek	1970	20	2	2 (100)	1 (50)	18	30 (83)	16 (44)	1.9 ± 0.3
	1971	61	15	6 (40)	5 (33)	46	74 (80)	29 (32)	1.8 ± 0.4
	1972	61	13	8 (61)	6 (46)	48	64 (67)	30 (31)	1.8 ± 0.4
	1973	61	22	14 (64)	11 (50)	39	53 (68)	20 (26)	1.6 ± 0.5
	1974	68	18	12 (67)	9 (50)	50	86 (86)	42 (42)	1.7 ± 0.4
	1975	87	14	10 (71)	9 (64)	73	112 (77)	44 (29)	1.8 ± 0.4
	1976	67	10	6 (60)	4 (40)	57	100 (88)	45 (39)	1.9 ± 0.4
	Total	425	94	58 (62)	45 (48)	331	519 (78)	226 (34)	1.8 ± 0.4
Manmanning	1969	4	0			4	5 (63)	2 (25)	2 ± 0
	1970	15	5	2 (40)	0	10	16 (80)	6 (30)	1.7 ± 0.5
	1971	21	4	1 (25)	0	17	25 (74)	9 (26)	1.8 ± 0.4
	1972	14	6	3 (50)	1 (17)	8	13 (81)	0	1.6 ± 0.5
	1973	16	7	3 (43)	2 (29)	9	12 (67)	2 (11)	1.6 ± 0.5
	1974	12	4	2 (50)	1 (25)	8	16 (100)	5 (31)	1.7 ± 0.5
	1975	12	4	2 (50)	1 (25)	8	12 (75)	4 (25)	1.7 ± 0.5
	1976	4	1	0	0	3	4 (67)	2 (33)	1.8 ± 0.4
	Total	98	31	13 (42)	5 (16)	67	103 (77)	30 (22)	1.7 ± 0.5
Tarwonga	1969	8	2	1 (50)	1 (50)	6	7 (58)	4 (33)	1.8 ± 0.4
	1970	15	3	1 (33)	1 (33)	12	19 (79)	7 (29)	1.8 ± 0.4
	Total	23	5	2 (40)	2 (40)	18	26 (72)	11 (31)	1.8 ± 0.4
Moornaming	1970	17	8	4 (50)	2 (25)	9	15 (83)	8 (44)	1.5 ± 0.5
Nereeno Hill	1975	8	4	1 (25)	1 (25)	4	6 (75)	0	1.5 ± 0.5
‘Midlands’	1996	21	7	6 (86)	4 (57)	14	14 (50)	8 (29)	1.8 ± 0.4
	1997	9	5	5 (100)	4 (80)	4	4 (50)	4 (50)	1.4 ± 0.5
	1998	9	4	4 (100)	3 (75)	5	6 (60)	4 (40)	1.5 ± 0.5
	Total	39	16	15 (94)	11 (69)	23	24 (52)	16 (34)	1.6 ± 0.2

The studies at the different localities were conducted by different researchers with different objectives: D.A.S. at Coomallo Creek 1969–1996, Manmanning, Tarwonga, Moornaming and Nereeno Hill; P.R.M. at Moora and Koobabbie 1996–1998 and P.R.M. and R.D. at Moora and Koobabbie 2003–2012, Borden, Kwobrup and Badgebup and Cocanarup 2008–2012 and Coomallo Creek 2003; and D.A.S., P.R.M. and R.D. at Coomallo Creek 2009–2012. Accordingly, the amount of time devoted to studying the birds in each locality differed markedly (Supplementary Table 1) and the amount of data generated varied. At Coomallo Creek and Manmanning, studies on Carnaby's Cockatoo were intensive and attempts were made to find as many breeding attempts as possible. At the other locations, as many breeding attempts were found as were possible in the limited time available. However, in each location the contents of nest hollows in which Carnaby's Cockatoo were breeding were noted wherever they were accessible by ladder.

### Clutch size, hatching success and fledging success

Clutch size, hatching success and fledging success for each active nest hollow could not always be established with certainty because there were not enough visits made in some years. Hence, clutch sizes, hatching success and fledging success for Coomallo Creek each year from 1977, Tarwonga 1971, Moornaming 1969, Nereeno Hill 1974, 1977–1980 and ‘Great Southern’ are not available.

Fledging success is defined here as the percentage of eggs produced that give rise to nestlings that successfully leave the nest hollow, while nesting success is defined here as the percentage of nesting attempts that produce at least one fledgling. That is, one pair producing one fledgling from a single-egg clutch constitutes one successful breeding attempt, as do one pair producing one or two fledglings from a two-egg clutch. Nesting success and fledging success are the same for single-egg clutches, but not for two egg clutches, where one nestling fledging is a successful breeding attempt. In this case nesting success equals 1 but fledging success equals 0.5.

### Ageing of nestlings and estimation of laying dates

In most cases any nestlings large enough to be handled were leg-banded (ringed) with Australian Bird and Bat Banding Scheme size 21 stainless steel-leg bands, the length of their folded left wing was measured (to the nearest millimetre) and they were weighed (to the nearest 10 g). Where nestlings were measured, they were aged using the length of their folded left wing compared against a growth curve of folded left wing generated from nestlings of known age at Coomallo Creek as described by Saunders (1986). This population was regarded by Saunders (1986) as the one in which the nestlings were in the healthiest condition. The accuracy of ageing nestlings using this method was ±4–6 days. The accuracy of ±4 days applied to nestlings aged about 31 days old and ±6 days applied to nestlings about 64 days old. This is a 13% variation at 31 days and 9% at 64 days. This is a reasonable error over a nestling period of more than 70 days. By ageing nestlings using this method, the dates when the eggs were laid and hatched were extrapolated. In the ‘Midlands’ during 1996–1998 no nestlings were handled. In these cases, the ages of the nestlings were estimated based on the researchers' experience of Carnaby's Cockatoo nestlings. These initial estimates of age were subsequently validated against their fledging dates.

### Assessment of nestling condition

Saunders (1982) developed a growth curve for Carnaby's Cockatoo of the relationship between estimated age and expected body weight with confidence intervals that ranged from ±22.2% of body weight for nestlings 11–15 days old to ±4.7% of body weight for nestlings 76–80 days old. By comparing the observed body weights of nestlings with the expected weights relative to the estimated ages based on the measurement of the folded left wing it was possible to determine which nestlings were in poor condition. We took poor condition as being defined by those nestlings whose body weight was more than 1 SD below the expected mean body weight for their estimated age (hereafter referred to as being below benchmark). Measurements of the folded left wing and weight were taken from both nestlings of 61 sets of siblings at Coomallo Creek from 1970 to 2012, making it possible to age each nestling and assess their weight against the benchmark; any falling more than 1 SD below the mean weight for the area were deemed to be ‘at risk’ (Saunders, 1986).

### Identification of individuals

Between 1971 and 1976 at Coomallo Creek and Manmanning the adults and large nestlings were marked with numbered leg bands (rings) and with a patagial tag with a two-letter combination (Saunders, 1988). As a result of problems associated with the use of patagial tags, from 1976 birds were individually marked with leg bands only.

### Estimation of the extent of native ­vegetation remaining in study locations

Carnaby's Cockatoo now breeds in hollows in eucalypt trees in patches of remnant vegetation on private property, in farm paddocks, on conservation reserves (Saunders, 1979b; Saunders *et al.*, 1982) and road and rail reserves. During the breeding season it depends mainly on seed and insect larvae obtained from native vegetation (Saunders, 1980; Department of Environment and Conservation, 2010). Using geographic information systems (GIS) and Western Australian government data sets on the extent of remnant native vegetation throughout the wheatbelt of south-western Australia, detailed localized maps of the extent of remnant native vegetation within a 6 and 12 km radius of individual nest hollows within each of the 10 locations were generated (Fig. 1). These distances were chosen because Saunders (1980) showed that breeding birds usually foraged within 6 km of their nest hollows, but they occasionally foraged within a radius of 12 km. Calculations were then made to determine the percentage loss of native vegetation within those polygons. No attempt was made in any of the study locations to estimate the relative quality of the remnant native vegetation with respect to its capacity to provide food for Carnaby's Cockatoo during the breeding season.

### Examination of nesting success in ­relationship to loss of native vegetation

Saunders (1986) examined breeding season, nesting success and nestling growth and assessed the viability of Carnaby's Cockatoo populations at Coomallo Creek, Manmanning, Tarwonga, Moornaming and Nereeno Hill. He demonstrated that this method for assessing viability could be applied more generally. Using the same populations, Saunders and Ingram (1987) examined nesting success in relationship to the extent of loss of native vegetation. With the benefit of more recent survey work in the same locations (Coomallo Creek, Nereeno Hill, Moornaming and Tarwonga) and Koobabbie and the application of GIS technology, it was possible to revisit those analyses with specific reference to the amount of native vegetation within a radius of 6 and 12 km of the nest sites.

### Sexing of nestlings

From many years of field experience we have found that Carnaby's Cockatoo nestlings can be sexed reliably from 4 weeks of age on the size and colour of the cheek patch. Males have cheek patches that are smaller and a dirtier white colour than females. The accuracy of this method has been verified recently by DNA testing of epithelial cells attached to breast feathers collected from nestlings in 2009 at Coomallo Creek, and from ‘Midlands’ and ‘Great Southern’ in 2008 and 2009 using the methods of White *et al.* (2009).

### Statistical analyses

The data set generated from this study has some unusual aspects that influence the types of analyses that can reasonably be performed. The study spans a 44 year period, with data collected from one or more sites in 34 of those years. The data set from one site (Coomallo Creek) is considerably larger (*n* = 26) than for any other site. The focus of the study is on fledging and nesting success in relationship to clutch size and the extent of remaining native vegetation in the immediate area around the study sites. At first glance a generalized linear modelling type analysis would seem appropriate, but there are a large number of missing data cells in the data set, which would devalue the results and make interpretation difficult. A repeated-measures style of analysis is also difficult to apply with any degree of certainty given what is known about the breeding biology of Carnaby's Cockatoo with regard to how individual females respond to failure of clutches at various stages of incubation and rearing of nestlings, and how that influences any attempts to relay in the same season (Saunders, 1982). It is also difficult to determine what the unit of any repeated measures would be. Given the small number of adult females that could be identified in the field (via leg bands) there is no capacity to use females as the ­measure, and also given the long time span at Coomallo Creek it is unlikely that the same females would have been alive across that time span. We do know from one of the study sites (Koobabbie) that events such as disease outbreaks removed breeding females from the population in two of the years when the site was studied (Saunders *et al.,* 2011). If the metric for repeated measures is changed from the individual breeding female to the nest hollow used (these have been clearly marked throughout the life of the study), it is still not appropriate to use this type of analysis because factors not related to the breeding birds influence nest hollow availability (nest competitors; flooding of the hollow; changes in depth to the chamber floor; loss of individual trees due to storm damage, tree fall, clearing and wildfire) and mean that year to year the number of nest hollows available to the cockatoos varies at each site, as does the make-up of the suite of hollows used within any single year.

With regard to the measure of health we provide for nestlings (see ‘*Assessment of nestling condition*’ above; Saunders, 1982, 1986), the standard error in those measurements varies according to the nestling's age and is smaller at the earliest and latest stages of the nestling period. Conducting an analysis on a continuous measure of body condition becomes more difficult. The large number of nestlings for which we have data together with the bias in favour of nestlings from Coomallo Creek make this type of analysis less useful. Our interest is primarily in nestlings that are underweight and which might be at risk of increased mortality. Despite the large number of nestlings that have been leg banded in this study (*n* > 1000) there have been too few band recoveries to provide enough data to inform any discussion on the relationship between nestling body weight and any particular benchmark and future survival of underweight chicks.

Accordingly, we have chosen to use simple but robust methods of analysis (Pearson's χ^2^, Fisher's exact test and simple liner regression) to describe the significance of observed relationships set out in this study.

## Results

### Clutch size

Clutch sizes for Coomallo Creek (1970–1976), Manmanning (1969–1976), Tarwonga (1969–1970), Moornaming (1970), Nereeno Hill (1975) and ‘Midlands’ (1996–1998) are shown on Table 1. Two eggs constituted the most common clutch at Coomallo Creek (78% of all clutches), Manmanning (68%), Tarwonga (78%) and ‘Midlands’ (59%). Limited data were available for only one season each at Moornaming and Nereeno Hill, and in these areas half of the total clutches were of two eggs. The clutch size of 610 nesting attempts in these locations was known, and the average clutch size was 1.7 ± 0.4.

### Hatching success

In all locations except the ‘Midlands’, hatching success (i.e. the percentage of eggs laid that hatch) for two-egg clutches was higher (50–100%) than for single-egg clutches (25–71%; Table 1). In the ‘Midlands’ hatching success of single-egg clutches was higher in all 3 years. Over all areas, in 77% of two-egg clutches both eggs hatched.

### Fledging success

Fledging success was significantly lower (χ^2^ = 49.3, d.f. = 12, *P* < 0.001; Table 1) for clutches of two eggs than for one at Coomallo Creek (34% for two-egg clutches compared with 48% for one), Tarwonga (31 cf. 40%) and ‘Midlands’ (34 cf. 69%), but higher at Manmanning (22 cf. 16%) and Moornaming (44 cf. 25%).

### Nesting success

Nesting success was significantly greater (χ^2^ = 47.2, d.f. = 12, *P* < 0.001; Table 1) for attempts with clutches of two rather than one: Coomallo Creek 66% (range 51–83%) for two-egg clutches compared with 48% (33–64%) for one; Manmanning 44 (0–67%) cf. 16% (0–29%); Tarwonga 61 cf. 40% (data for two seasons only); Moornaming 89 cf. 25% (one season only); but in the ‘Midlands’ single-egg clutches had the same nesting success as two-egg clutches (69 cf. 70%).

### Clutch size of individual females

The clutch sizes of 26 individually marked females from Coomallo Creek and eight from Manmanning were established over at least three breeding seasons and up to 10 seasons for one female. Only one of these was a female of known age. MT (patagial tag) fledged at Coomallo Creek in 1969 and commenced breeding at age 4  years (the minimal age for breeding; Saunders and Ingram, 1998). In her first breeding year (1973) she laid a clutch of two eggs, one the next year and made two unsuccessful breeding attempts in her third breeding year, each of two eggs in different nest hollows. The remaining 25 females at Coomallo Creek were individually marked when they were adults, so they were at least 4 years old when marked. Thirteen produced only clutches of two eggs over observation periods of 3 (six individuals), 4 (three individuals), 5 (one individual) and 6 years (three individuals). Of these females, four bred again in a different hollow the same season after an earlier breeding attempt had failed. Each produced clutches of two eggs in the second attempt; two were successful at fledging a nestling with the second breeding attempt. With the other 12 females there was no pattern in the order that clutches of different size were produced. For example, seven females produced a single-egg clutch in the first year of observation and, of the other five females, three produced a single-egg clutch the second year of observation, one in the third year and one in the fourth. None of the females at Coomallo Creek produced only ­single-egg clutches over the period when they were under ­observation.

Of the eight females at Manmanning, two produced only single-egg clutches; one over 3 years and the other over 4 years. Four females produced only clutches of two eggs; one over 3 years and the others over 4 years. Of the remainder, one over a 5 year period produced four clutches of two eggs and a single one-egg clutch in the second year of observations. The other, over a 4 year period produced three clutches of two eggs, while in the third year of observation she had two unsuccessful nesting attempts in different hollows, each of one egg.

### Clutch size in relationship to loss of native vegetation

There was a trend of a decreasing clutch size with a decreasing percentage of remnant native vegetation in the localities surrounding nesting sites, but the relationship was not ­significant (*r* = 0.70, d.f. = 4, 0.05 < *P* < 0.10).

### Nesting success in relationship to loss of native vegetation

**Figure 2:**
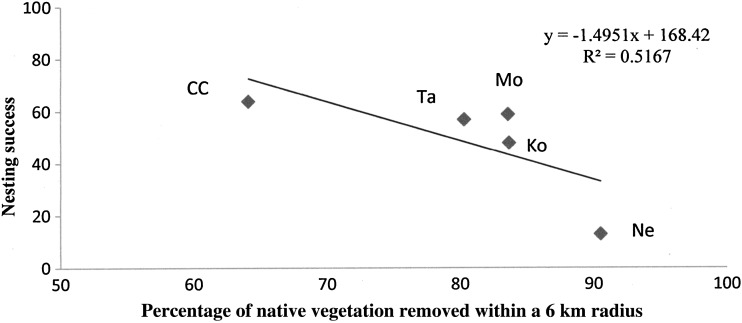
Nesting success (percentage of nesting attempts producing at least one fledging) in relationship to percentage of native vegetation removed from within a 6 km radius around Coomallo Creek (CC), Koobabbie (Ko), Moornaming (Mo) Tarwonga (Ta) and Nereeno Hill (Ne).

There was a strong, but not significant, negative correlation between nesting success and the percentage of native vegetation cleared within a 6 km radius of nest sites (Fig. 2; *r* = 0.72, d.f. = 4, 0.05 < *P* < 0.10). However, there was no significant relationship between nesting success and percentage loss of native vegetation up to 12 km from nest sites (*r* = 0.41. d.f. = 5, *P* > 0.10).

### Incidence of siblings surviving for 14 days or more

The incidence of both nestlings (referred to hereafter as siblings) from a clutch of two eggs surviving for at least 14 days at Coomallo Creek, Manmanning, Tarwonga, Moornaming, ‘Midlands’ and ‘Great Southern’ is shown in Table 2. In each location, except Nereeno Hill, at least 4% of clutches of two eggs had siblings survive for more than 14 days. However, no clutches at Manmanning or Moornaming resulted in both nestlings fledging. There were insufficient visits to the ‘Great Southern’ during 2008–2012 to determine whether both nestlings fledged.

**Table 2:** Incidence of siblings from a breeding attempt surviving for more than 14 days and incidence of both nestlings fledging

Year	Coomallo Creek	Manmanning	Tarwonga	Moornaming	Nereeno Hill	‘Midlands’	‘Great Southern’
1969	11/0/0	6/0/0	13/1/0	7/0/0			
1970	29/1/1	18/0/0	15/3/0	18/1/0			
1971	64/1/0	23/3/0	5/2/0				
1972	74/2/2	14/1/0					
1973	77/1/0	16/0/0					
1974	78/2/2	13/1/0					
1975	91/2/2	12/1/0			8/0/0		
1976	69/2/1	6/0/0			2/0/0		
1977	40/1/1				2/0/0		
1978	38/2/?				3/0/0		
1979					4/0/0		
1980					2/0/0		
1981	38/9/0						
1982	46/2/?						
1983	41/6/?						
1984	39/2/?						
1985	46/4/?						
1986	52/4/?						
1988	47/3/?						
1989	44/2/?						
1990	53/2/?						
1994	32/2/?						
1996	37/1/?					21/1/0	
1997						9/1/1	
1998						9/1/0	
2003	7/0/0					31/0/0	
2005						10/0/0	
2006						14/0/0	
2007						17/0/0	
2008						19/1/?	26/5/?
2009	41/6/6					13/0/0	13/2/?
2010	49/5/5					9/0/0	17/3/?
2011	53/2/2					6/0/0	15/4/?
2012	76/6/6					8/0/0	5/3/3
Total	1272/70/?	108/6/0	33/6/0	25/1/0	21/0/0	166/4/?	76/17/?

The first number is the number of breeding attempts. The second number is the number of nests with siblings. The third number is the number of nests where both siblings fledged. ‘?’ indicates that no data are available on fledging history for that season.

Two populations in the southern wheatbelt had higher rates of siblings surviving to 14 days or more (Tarwonga 18.1% and ‘Great Southern’ 17.9%) compared with the northern sites (Coomallo Creek 4.3%, Manmanning 5.6%, Nereeno Hill 0% and ‘Midlands’ 6.8%). Moornaming, with a rate of 4.0%, was similar to locations in the northern wheatbelt. Although the ‘Great Southern’ was visited only once each year, the observations of two surviving nestlings are valid because when visits were made, both nestlings were old enough to have leg rings applied, something that is not possible to do safely until nestlings are at least 3 weeks old.

### Difference in age between siblings

**Figure 3:**
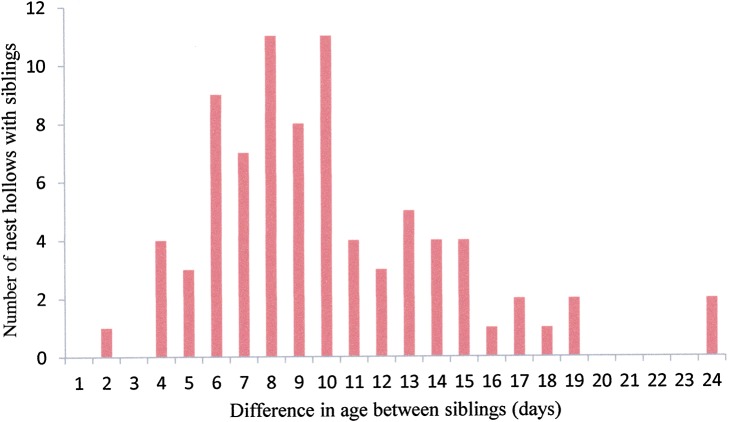
Difference in age (in days) between siblings from all locations studied.

**Figure 4:**
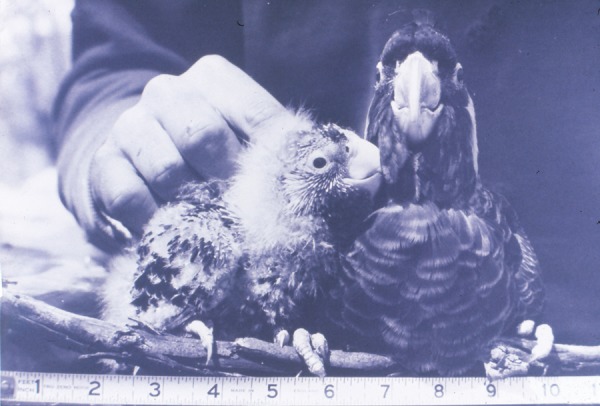
A set of siblings from a nest hollow at Tarwonga in November 1969. The older nestling is 44 days old and the younger 28 days. The scale is in inches. (Photograph. D. A. Saunders.)

Female Carnaby's Cockatoo begin incubation with the laying of the first egg, and both eggs have an incubation period of 28–29 days (Saunders, 1979c, 1982). Accordingly, eggs hatch asynchronously, with the same interval as between laying. Examining the 55 Coomallo Creek, five Manmanning, six Tarwonga, one Moornaming, one ‘Midlands’ and seven ‘Great Southern’ sets of siblings for which measurements were available, the difference in age between them ranged from 2 to 24 days (Figs 3 and 4), with the median difference of 9 days considering all sets of siblings. The median difference was 9 days at Coomallo Creek, 8 days at Manmanning, 10 days at Tarwonga and 9 days in the ‘Great Southern’. The only sets of siblings measured at Moornaming and ‘Midlands’ each had a difference in age of 10 days.

### Laying dates for clutches with siblings in relationship to the commencement of egg laying for the season

**Figure 5:**
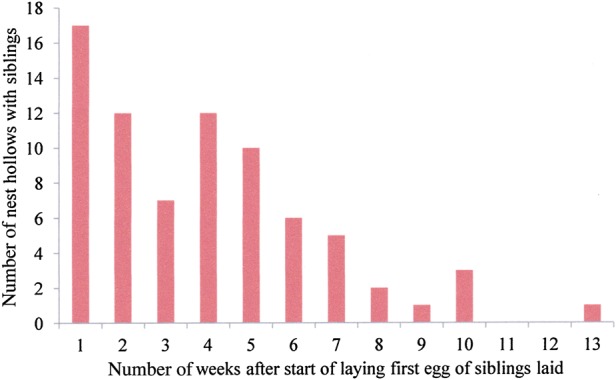
Timing of laying of the first egg in relationship to the commencement of egg laying that season in nests where both nestling survived for at least 14 days. Week 1 is the week in which egg laying commenced at that location in any year.

Laying dates for the first egg for each set of siblings at all areas ranged from the commencement of the egg-laying season until week 13 of the season (Fig. 5), with a median of week 4 after the commencement of egg laying. At Coomallo Creek the range was from the start of the laying season until week 10, with the median of week 4. For those 19 sets of siblings at Coomallo Creek where nesting outcome was known, both nestlings fledged from eggs laid from week 2 to 8 after the start of the egg-laying season. At Manmanning, clutches with siblings were commenced from week 1 after the commencement of the laying season until week 4. The range at Tarwonga was from week 1 until week 5, and in the ‘Great Southern’, from week 1 until week 13. There was a marked trend (76.3%) for the production of siblings to occur in the first half of the egg-laying season (Fig. 5).

### History of known females raising siblings

The breeding history of eight individually marked females at Coomallo Creek known to have produced siblings is shown in Table 3. The number of known breeding attempts made by these females ranged from two to 12. Two of these females were banded as nestlings in the area and so were of known age. One raised siblings at 17 years old and the other at 20 years old. The other six females were banded as breeding adults and so were at least 4 years old when banded. One was at least 5 years old when she produced siblings and the others were at least 9 (two females), 14, 22 and 26  years old. Three of these produced more than one set of siblings in the period during which they were observed breeding.

**Table 3:** Breeding history of individually marked females at Coomallo Creek known to have fledged both nestlings successfully from one breeding attempt

Female	1970	1971	1972	1973	1974	1975
210-00023	4 + /2/1/wk6	5 + /?/1/wk4	6 + /2/1/wk4	7 + /1/1/wk4	8 + /2/1/wk5	**9 + /2/2wk3**
210-00346				4 + /1/1/wk3	5 + /1/0/wk6	6 + /2/0/wk6
					5 + /1/0/wk12	

Female	1976	1977	1981	1982	1983	1984
210-00023	**10 + 2/2/wk7**	11 + /2/1/wk4				
210-00346	7 + /2/1/wk5	8 + /2/0/wk6	12 + /2/1/wk2	13 + /2/1wk4	**14 + /2/2/wk2**	15 + ?/1/wk5

Female	1985	1986	1988	1989	1990	1994
210-00346	16 + /2/?/wk5	17 + /2/1/wk5				
210-03011	4 + /?/1/wk10	5 + /1/1/wk9		8 + /?/1/wk9	**9 + 2/2/wk8**	
210-03143	4 + /?/1/wk3	**5 + /2/2/wk5**	**7 + /2/2/wk4**	8 + /?/1/wk2		
210-01876			4 + /2/1/wk4		6 + /2/1/wk6	
210-03089						4 + /?/1/wk9
210-01422*	8/?/?/wk3					**17/2/2/wk6**

Female	2009	2010	2011	2012		
210-01876	25 + /2/1/wk1	**26 + /2/2/wk5**	**27 + /2/2/wk4**	28 + /2/1/wk8		
210-03089	19 + /2/1/wk5	20 + /?/0/wk8	21 + /2/0/wk10	**22 + /2/2/wk6**		
210-01694*	19/2/1/wk4	**20/2/2/wk1**	21/2/1/wk3	22/2/1/wk4		

The first number is age (in years); birds marked as adults have ‘ + ’ after year because they were at least 4 years old when banded and, therefore, the number indicates the minimum age that year. The second number is the number of eggs laid. The third number is the number of nestlings that fledged. The fourth number is the week (wk) in which the first egg of the clutch was laid. ‘?’ indicates that no information is available. Birds of known age are indicated by ‘*’ after their band number. Data in bold indicate both nestlings fledged.

### Health of siblings at Coomallo Creek, ‘Midlands’ and ‘Great Southern’

**Figure 6:**
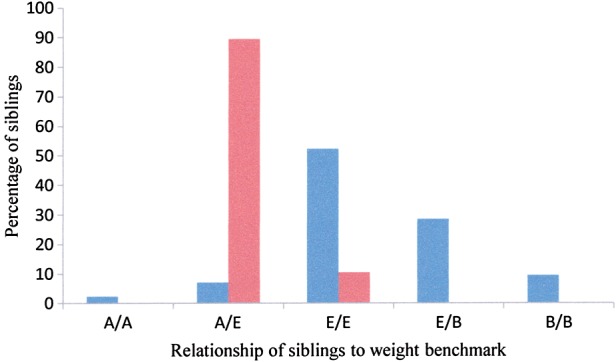
Percentage of sets of siblings at Coomallo Creek from 1970–1996 (blue, *n* = 42) and 2009–2012 (red, *n* = 19) in which both were above benchmark weight (A/A), one was above and the other equal to benchmark weight (A/E), both were equal (E/E), one was equal and one below (E/B) and both were below (B/B).

The measurements of 42 sets of siblings were known from 1970–1996 and 19 sets from 2009–2012. There was a marked significant difference in the health of siblings between these two periods (Fig. 6), with 61.9% having both nestlings equal to the benchmark, one equal to and the other above or both above the benchmark during 1970–1996 compared with 100% during 2009–2012. During 1970–1996, 38.1% of sets had one equal to and one below or both below the benchmark, compared with none in these categories during 2009–2012 (χ^2^ = 40.6, d.f. = 4, *P* = 0.000).

Twenty (16.4%) of the 122 nestlings had weights below the benchmark. Eight of these belonged to four sets of siblings. The fledging history of two of these sets was known and, in both cases, both nestlings fledged. Of the other 12 below the weight range, eight were the younger and smaller nestlings (at hatching) of the set, while the remainder were the older and larger nestlings. All of the 10 nestlings in five sets recorded from individually marked females had body weights above the benchmark.

Of 21 sets of siblings from 1970–1977 whose fledging success was known, 42.8% were successful in fledging both nestlings. This is considerably less than the 100% for the 19 sets from 2009–2012 (Table 2).

If the growth curves derived for the Coomallo Creek site (Saunders, 1986) are used as a benchmark, it is possible to compare the health of nestlings produced at the other locations relative to those of Coomallo Creek. The younger of the nestlings from the set of siblings at the ‘Midlands’ in 2008 had a measured weight below the Coomallo Creek benchmark. Only one chick from the five sets from the ‘Great Southern’ recorded in 2008 was below the benchmark and, again, it was the younger of the nestlings. None of the nestlings in the 12 sets recorded in the ‘Great Southern’ during 2009–2012 was below the benchmark.

### Health of single nestlings at Coomallo Creek

In the period 1970–1996 at Coomallo Creek, 681 single nestlings were measured and aged, and 156 during 2009–2012. There were no differences in the health of single nestlings between the two periods. During 1970–1976, 10.6% were above benchmark for weight, 79.3% were equal to the benchmark and 10.1% were below, compared with 14.1% above, 78.2% equal and 7.7% below during 2009–2012 (χ^2^ = 0.89, d.f. = 2, *P* = 0.64).

When the number of siblings that were produced relative to the number of single nestlings at Coomallo Creek during 1969–1977 and 2009–2012 are examined, there is a significant difference (in favour of the later period; χ^2^ = 53.91, d.f. = 12, *P* = 0.001). This suggests that despite the continued loss of remnant vegetation (∼10%) during the intervening years, the cockatoos have managed to find a suitable alternative food to sustain and even improve reproductive output.

### Health of nestlings in different localities in relationship to percentage of native vegetation removed from the landscape

**Figure 7:**
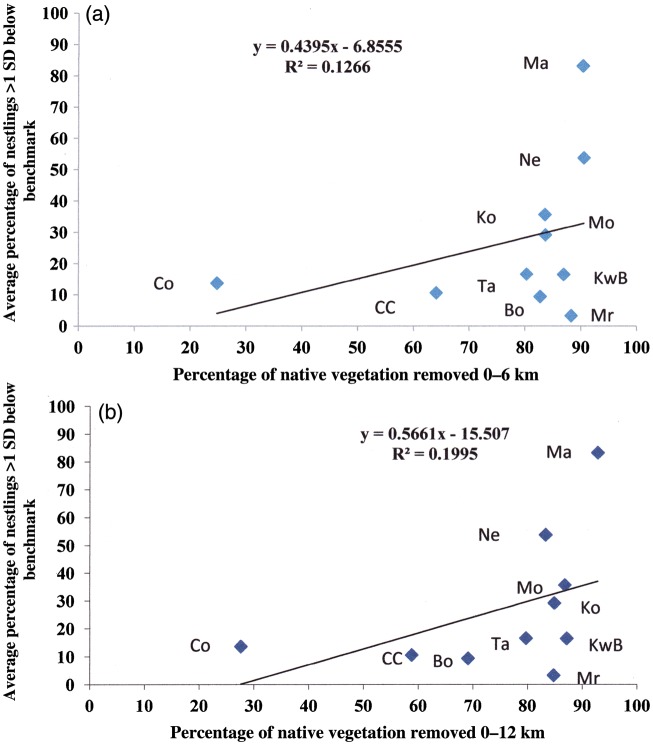
Percentage of nestlings below 1 SD of mean body weight for age relative to the percentage of native vegetation removed within a 6 km radius (**a**) and a 12 km radius (**b**) of nest hollows at Coomallo Creek (CC), Koobabbie (Ko), Nereeno Hill (Ne), Manmanning (Ma), Moora (Mr), Moornaming (Mo), Tarwonga (Ta), Kwobrup and Badgebup (KwB), Borden (Bo) and Cocanarup (Co) locations.

Using the relationship between age and predicted body weight developed from the Coomallo Creek data (Saunders, 1986), the health of nestlings from Coomallo Creek, Koobabbie, Nereeno Hill, Manmanning, Moora, Moornaming, Kwobrup and Badgebup, Cocanarup and Borden was examined. There was a positive but not significant relationship (*r* = 0.39, d.f. = 9, *P* > 0.10) between the percentage of all nestlings below benchmark weight for age and the percentage of native vegetation removed from within a radius of 6 km of nesting hollows (Fig. 7a). A similar but stronger relationship (*r* = 0.45, d.f. = 9, 0.05 < *P* < 0.10) was found between the percentage of all nestlings below benchmark weight for age and the percentage of native vegetation removed from a radius of 12 km of nesting hollows (Fig. 7b).

### Survival of individually marked siblings

Both siblings from only one set of siblings from Coomallo Creek were seen after they had fledged. There was 10 days difference in age, and they fledged in late 1974. Both were seen with their parents along the Hill River (16 km west of their nest site) several times between January and March 1975. The younger nestling was seen again, also along the Hill River in January 1976 and January 1977. One sibling of four sets was recorded after fledging. In each case their sibling was not seen after fledging. The younger nestling (by 8 days) from nest #39 in 1972 was taken by a Wedge-tailed Eagle (*Aquila audax*) soon after it fledged. The younger nestling (by 10 days) from nest #160 in 1974 was seen with its parents in the study area not long after fledging. The younger nestling (by 6 days) from nest #78 in 1975 was seen several times between February and March 1977 at King Ranch near Eneabba, 30 km northwest of Coomallo Creek. The only ­siblings known to have bred at Coomallo Creek were both younger nestlings. One was younger by 14 days from nest #15 in 1977. She was recorded breeding in 1985 when she was 8 years old and again in 1994 at 17 years old. The other was younger by 15 days from nest #106 in 2009 recorded breeding in 2013, 143 m from where she fledged.

### Sex of siblings

Twenty-four sets of siblings were sexed at Coomallo Creek from 1970 to 2012. Seven sets consisted of males only, eight sets were of females only and nine sets of males and females, an overall sex ratio of 25 females to 23 males; not a significant departure from parity. The two sets at Manmanning consisted of females in one and mixed sexes in the other. The one set at Tarwonga was mixed sex and the one set at Moornaming was of females. Of the nine sets at Coomallo Creek of males and females, in six of these the female was the older sibling. In the one mixed set at Manmanning and Tarwonga the female was the older sibling. In the ‘Midlands’ in 2008, the set of siblings consisted of a male and female, with the female being the older. At ‘Great Southern’ between 2008 and 2010 there were seven sets of siblings where the sexes were known. Four were of males only, one was of females only and two were mixed sexes; in both of these sets, the male was the older nestling. The sex ratio was 10 males to four females.

## Discussion

### Clutch size

There are interesting differences in the sizes of the clutches between those black cockatoos with red tail bands (Red-tailed and Glossy Black Cockatoo) and those with white and yellow tail bands (Carnaby's and Yellow-tailed Black Cockatoo). The former have only been recorded laying single-egg clutches and the latter usually produce clutches of two eggs. Why produce two eggs in a breeding attempt when only one nestling is usually raised successfully? It has been postulated that in birds, clutch size is an adaptation to the largest number of young for which parents can provide enough food (Lack, 1954). Ehrlich *et al.* (1988) pointed out that some birds usually lay more eggs than they can successfully rear and cite the example of Whooping Cranes (*Grus americana*), which usually produce clutches of two eggs, but almost always only rear one young. Brooker (1976) and Ridpath and Brooker (1986) noted that Wedge-tailed Eagles usually rear one young from clutches of two, but in good seasons they can rear two. Ehrlich *et al.* (1988) suggested that as it takes relatively little energy to produce the second egg, it acts as insurance in the case of the first failing. The second egg certainly provides insurance in the event that the first egg does not hatch or the first nestling dies before the second egg hatches. Unfortunately, we have no data on the incidence of the second egg giving rise to a successful fledgling after the failure of the first egg or nestling in Carnaby's Cockatoo because we did not mark the eggs. However, the fact that there are occasions where the second egg has given rise to a successful fledgling indicates that it is a useful strategy to produce two eggs.

Saunders *et al.* (1984) collected data on body weights and egg weights of 54 taxa of Australian parrot (Saunders *et al.,* 1984, their Table 1). The species ranged in size from the 37 g Scarlet-chested Parrot (*Neophema splendida*), which lays four to seven eggs, each of which is about 10.8% of the female's body weight, to the Palm Cockatoo; 760 g), whose one egg weighs about 4.9% of the female's body weight. Carnaby's Cockatoo eggs weigh about 31 g (Saunders *et al.*, 1984), which is 4.8% of the body weight of the female (Saunders *et al.*, 1984; Table 1). The eggs of the Yellow-tailed Black Cockatoo are a similar percentage of the females' body weight. Interestingly, the eggs of the Red-tailed Black Cockatoo, which lays only one egg, were about the same percentage of the females' weight (4.8%) as those of Carnaby's Cockatoo, but those of the Glossy Black Cockatoo were 6.1% of the females' body weight. The physiological burden on female black cockatoos of producing eggs is not large compared with that of the smaller parrots, for which individual eggs may be over 10% of the body weight of the females and where up to seven eggs may be laid in a clutch. In the case of Carnaby's Cockatoo, where the second egg is usually produced a week later (Fig. 3), the female has the opportunity to build up her reserves for the production of the second egg.

Saunders (1982) noted that at Coomallo Creek, out of 222 nests where sufficient records were available to monitor the fate of both the eggs, in only 11 cases where both eggs hatched did the second nestling survive for more than 3 days. Saunders (1982) recorded that an adult Carnaby's Cockatoo that has lost its mate is capable of successfully fledging a nestling. As shown in the present study, the species is capable of successfully fledging both nestlings from a two-egg clutch. Why, therefore, does the second hatchling usually die so soon after hatching? There are several possibilities. One is lack of maternal care. Another is aggression from the older nestling. While we have examined many nest hollows, our presence resulted in sitting females vacating their hollows, and we were unable to observe incubating and ­brooding behaviour. However, we know of information on the maternal behaviour of one breeding female. During the cockatoo breeding season of 2010 at Coomallo Creek, Leighton De Barros (Sea Dog Productions) collected material to produce a film of the annual cycle of Carnaby's Cockatoo. He installed a camera in the floor of one active nest hollow and recorded nesting activity within the hollow for part of nearly every day from preparation of the hollow floor by a Carnaby's Cockatoo female to fledging of the nestling. Much of the film was streamed to the worldwide web (http://www.wingandaprayer.com.au/). Of particular interest here is the attention the female paid to the hatching of each of her two eggs. As the first nestling hatched the female remained over the egg, had her bill touching the emerging nestling and fed it soon after hatching. The second nestling hatched a week later. The female left the hollow as this egg was hatching and provided no assistance to the hatchling (http://www.youtube.com/user/Onawingandaprayer1#p/u/64/_CQ-F0JRkkI accessed 8 July 2011). That nestling died within 24 h.

Sibling aggression leading to death or siblicide (Mock, 1984) is not uncommon in birds, occurring in a wide range of groups, including, but not restricted to, boobies (*Sula* spp.; Dorward, 1962; Anderson, 1990), eagles (*Aquila* spp.; Edwards and Collopy, 1983) and kingfishers (*Dacelo novaeguineae*; Legge, 2000). Saunders (1982) pointed out that asynchrony in hatching dates means that the older nestling is usually much larger than the younger one (Fig. 4). He recorded instances of the older nestling lunging at the younger one as it commenced begging. This behaviour may have resulted in the older nestling obtaining more food than the younger, with it starving. In fact, in the film sequence cited above, the older nestling can be seen lunging at the younger one as it is trying to emerge from the egg, and it is possible that this behaviour physically damages the younger nestling. Either maternal neglect or sibling competition may explain the loss of the second nestling in Carnaby's Cockatoo.

Studies of individually marked females did not reveal any pattern of clutch size in relationship to age of the breeding female. The only female of known age, with information from several breeding years, was at Coomallo Creek, and she produced a clutch of two eggs on her first breeding attempt. The fact that females at Manmanning had smaller clutches, did not rely on their mate for food while incubating and brooding young nestlings (Saunders, 1982), that some females there only ever produced single-egg clutches, when none did so consistently at Coomallo Creek, and females relied completely on their mate for food during incubation and brooding young nestlings indicates that food availability is the most likely influence on clutch size. The dependence of Carnaby's Cockatoo on native vegetation for food during the breeding season explains why average clutch size tends to decrease as the amount of native vegetation around breeding sites decreases (Fig. 7). Jetz *et al.* (2008) analysed the variation in clutch size in 5290 species of bird and found that seasonality of food resources was the predominant driver in variation in clutch size. Where food is limiting, clutch sizes are smaller. This would suggest that if further clearing occurs or if the quality of the remaining native vegetation declines either in response to local threats, such as salinity, altered hydrology and fire, or in response to climate change, then average clutch size may decline to the point where clutches of one may become the norm throughout the breeding range of Carnaby's Cockatoo.

### Incidence of sets of siblings

There are no published records of sets of siblings being fledged by Yellow-tailed Black Cockatoo or Baudin's Cockatoo. Sindel and Lynn (no date) noted that with the Yellow-tailed Black Cockatoo, the second chick is neglected and allowed to die, although on rare occasions two chicks are fledged. This statement is almost identical to an earlier statement on the subject by Forshaw (1981). This is the result of confusion of taxonomy of that time, when Carnaby's Cockatoo was regarded as a subspecies of Yellow-tailed Black Cockatoo. Carnaby's Cockatoo remains the subject of the most detailed ecological studies of the yellow/white-tail black cockatoo super-species, and it is the only species recorded as fledging both nestlings. The apparent unique nature of Carnaby's Cockatoo may simply be due to a lack of detailed field studies of Baudin's and the Yellow-tailed Black Cockatoo. The only other record we could find regarding siblings for Carnaby's Cockatoo related to the 2008 breeding season. D. Stojanovic (http://birdlife.org.au/documents/CBC-cockynotesmar09.pdf, accessed 7 February 2014) reported that five sets of siblings were reared to at least 8 weeks of age in the northern wheatbelt. The history of two sets of siblings was known. In one set there was a 2 week difference in fledging date between the siblings. In another set the elder nestling fledged, but the younger did not. No data were provided on the total number of active hollows studied or the area of the study, so no comparisons may be made between our data and those of Stojanovic.

What is apparent from our results is that sets of siblings are not uncommon in areas where food does not appear to be affecting the ability of the parents to raise healthy chicks. For example, at Manmanning, where food was demonstrably limited and impacting on the population (Saunders, 1982), there were no incidences of both nestlings being successfully fledged, in comparison to Coomallo Creek (Table 2). Of the 14 years at Coomallo Creek when fledging success was known, siblings contributed from 0 to 14.6% to the total number of fledglings per season (Table 2). It is reasonable to assume that fledging of both nestlings would result in a better recruitment rate than for cockatoo species that produce only single-egg clutches. This improved recruitment rate when combined with an original access to large expanses of native vegetation supporting proteaceous plant species that provide large and nutritious seeds would explain why Carnaby's Cockatoo was once so numerous (Perry, 1948). However, with 18 million hectares of habitat cleared for farming and that habitat which remains now fragmented, a predisposition to laying two eggs and rearing both nestlings might in fact result in reduced recruitment from nests with two eggs or nestlings. The increased incidence of nestlings with below-average body weights and the low or non-existent levels of sets of siblings at sites on the north-eastern margins of the distribution of Carnaby's Cockatoos (e.g. Nereeno Hill, Koobabbie and Manmanning; Table 2) suggest that breeding populations at sites such as these are doomed to local breeding extinction.

The difference in age between siblings ranged from 2 to 24 days, with a median difference of 9 days. This is similar to the 1–16 day range recorded for all clutches of two eggs reported by Saunders (1982). The fact that breeding pairs of Carnaby's Cockatoo can successfully raise two nestlings with such large differences in age is an indicator of the potential of their rearing skills and dedication to what can amount to an extended breeding effort in order to continue to feed and care for a second nestling while the older chick has fledged.

At Coomallo Creek, the timing of commencement of laying of sets of siblings did not seem to affect the production of siblings, as siblings were produced from the start to the end of the egg-laying period, although there was a clear bias towards sets of siblings coming from clutches laid in the first 5 weeks of a laying season (Fig. 5). Studies of individually marked females at Coomallo Creek indicate that sets of siblings are produced by older, and presumably more experienced, females. These females, together with their partners, were just as capable of raising two nestlings at once within the same healthy weight range as with their single-nestling breeding attempts. Another implication arising from these results is that, given that the incidence of sets of siblings appears to be associated with older and more experienced breeding females, our data may actually be showing that the age demographics of the breeding females is becoming skewed in favour of older birds. This may suggest that young females are not recruiting into the breeding population at the same rate as they once did. This hypothesis can only be tested by surveying the existing population for birds with leg rings, and even longer-term monitoring to determine the rate at which birds banded in the last decade return to their natal (or some other) breeding site.

### Sets of siblings as an indicator of adequacy of available food

Sets of siblings provide an indicator of the adequacy of food availability to populations of Carnaby's Cockatoo. At Coomallo Creek between 1969 and 1977, sets of siblings contributed 1.7% of the fledglings produced. Between 2009 and 2012, sets of siblings contributed nearly five times more to the population of fledglings (8.2%; Table 2). While the extent of native vegetation at Coomallo Creek has decreased from 90% in the 1960s to 35%, latterly there has been a major increase in the incidence of sets of siblings at Coomallo Creek, an increase in the successful fledging of both nestlings in a set and an increase in the contribution they make to the total number of fledglings. This seems anomalous. Until 1996, birds raising fledglings were completely dependent on native vegetation for food. However, since the late 1990s Canola (*Brassica napa* and *B. juncea*) has been grown as a commercial crop in the study area. Carnaby's Cockatoo started feeding on Canola seed immediately it was available (J. Raffan, personal communication; Jackson, 2009). We noted the birds feeding on Canola every day during mid-November surveys of the area every year from 2009 to 2012. One recently dead nestling found in a nest hollow in 2009 had a full crop, which was predominantly Canola seed along with several seeds of *Banksia* sp. Many other nestlings we have handled had round, brown seeds, clearly visible through the semi-translucent skin of their crops, suggesting that Canola was fed to most nestlings on a daily basis.

At Coomallo Creek the production of single nestlings below benchmark weight has not changed between 1970–1996 and 2009–2012, and sets of siblings have increased in number in the latter period. In addition, none of those nestlings hatched during 2009–2012 have been below benchmark (Fig. 6) despite the loss of remnant native vegetation, indicating the likely value of a replacement food such as Canola. We are of the view that Canola seed that is available during most of the nestling period has offset the loss of food from remnant native vegetation. Canola is now grown throughout much of the higher rainfall areas of the Western Australian wheatbelt, but the period of time that mature seed on standing crops and swathed crops is available to be fed to nestlings is shorter in the higher latitudes (southern wheatbelt) of the range of Carnaby's Cockatoo. In the southern wheatbelt the Canola crop has been harvested by the time the cockatoos are about 4–5 weeks from fledging their young. In these more southerly sites Carnaby's Cockatoo must rely either on spilt Canola seed in paddocks and along roadsides or on remnant native vegetation to raise nestlings ­successfully.

In a productive season, the second egg can provide a significant contribution to recruitment. By losing the ability to produce both nestlings from even a small proportion of nesting attempts, Carnaby's Cockatoo will have a potential net reduction of recruits to the juvenile population.

The importance of introduced species as food for breeding black cockatoos is shown by the case of the inland Red-tailed Black Cockatoo (*C. b. samueli*), which has expanded its range into the northern wheatbelt of Western Australia from the adjacent Murchison Region (Saunders *et al.*, 1985). In the both regions, Doublegee (*Emex australis*), an introduced weed species, has proliferated and forms the bulk of the bird's diet. The species is common where Doublegee is common and has both an autumn and spring breeding season; in some cases females have bred at 6 monthly intervals (Saunders, 1977). Doublegee is so important to the species that should an effective method of control be developed for the weed, the range and abundance of the species in agricultural areas would decline markedly. Introduced pines (*Pinus* spp.) are an important food source for Carnaby's Cockatoo during the non-breeding season (late January–March) and have been ever since the first commercial plantations started bearing cones (Perry, 1948; Stock *et al.*, 2013). Likewise, Aleppo Pine (*Pinus halensis*) has been identified as an important food source for Yellow-tailed black cockatoos (*C. funereus whitei*) living on the Eyre Peninsula in South Australia, and is thought to be the only thing sustaining this cockatoo population within its breeding range (Way and van Weenan 2008).

### Conservation implications

Geographic information system analyses show that nesting success is correlated with the amount of native vegetation within 6 km of the nest hollows and that the health of nestlings (as determined by the relationship between age and measured body weight) is correlated with the amount of native vegetation within 12 km of nest hollows (Fig. 7). We were unable to provide data on the quality of the remnant native vegetation; its retention in particular areas should not be taken as a guarantee of the continued breeding by Carnaby's Cockatoo in an area. Extensive and detailed habitat assessment, such as that undertaken by Johnston (2013) in parts of the non-breeding range of Carnaby's Cockatoo, will be required to define and refine that relationship further.

This long-term study, conducted over 44 years, has shown how Carnaby's Cockatoo has adapted to the extensive loss of native vegetation and accepted novel food sources. It has also shown that the previously recorded loss of breeding populations at the eastern and drier margins of its breeding range (Saunders, 1986, 1990) appears to be continuing, with key sites such as Koobabbie struggling to produce nestlings equal to or greater than benchmark weight. Given the suite of land-use changes (clearing for farming, mining and urban development) occurring in the western and coastal parts of its non-breeding range, the prognosis for the continued survival of this species in all of its current range appears bleak, although in those areas of its range where Canola is grown the populations may have better prospects.

The fate of the breeding populations of Carnaby's Cockatoo in the ‘Great Southern’ appears secure at present, but this may only be temporary, given the predictions for climate change in south-western Australia. Current predictive modelling of south-western Australia suggests further reductions in annual rainfall and increased frequency of drought, severe weather events and consequent fires arising from lightning (CSIRO, 2007; Hennessy *et al.*, 2008). Extreme weather events, including heat and hail, are already having an impact on Carnaby's Cockatoo (Saunders *et al.*, 2011). The changes associated with altered climate may make the growing of crops such as Canola more difficult or lead to a reduction in the area sown to Canola. It is also likely to lead to a loss of some species of endemic native plants with limited distributions, such as species of *Banksia*, *Hakea* and *Grevillea* (Steffen *et al.*, 2009), with detrimental impacts on Carnaby's Cockatoo. Pouliquen-Young and Newman (2000) assessed the effects of three climate scenarios (CSIRO, 1996) for 92 species of endemic Western Australian species of *Dryandra* (now included in the genus *Banksia*). The ­bioclimates for 55% of the *Dryandra* species were predicted to decline to less than half their current distributions, and 28% of species, all of which had current geographic ranges of less than 1000 km^2^, were predicted to disappear completely with a 0.5°C warming. With a 2°C warming, the bioclimates of 91% of the *Dryandra* species were predicted to decline by more than half their 2000 distributions and 66% to disappear completely. If a similar response to a warming climate were to be recorded in other species of Proteaceae (*Banksia*, *Grevillea*, *Isopogon*, *Petrophile* and *Lambertia*) then the impact on Carnaby's Cockatoo food supplies in some parts of the species' range could become ­significant.

## Supplementary material

Supplementary material is available at *Conservation Physiology* online.

Supplementary Data
