# Supplementary material for: One fledgling or two in the endangered Carnaby's Cockatoo (Calyptorhynchus latirostris): a strategy for survival or legacy from a bygone era?
Source: Conserv Physiol. 2014 Feb 17;2(1):cou001. doi: 10.1093/conphys/cou001 (PMC4732493; doi:10.1093/conphys/cou001)
Supplement: Supplementary Data [file supp_2_1_cou001__index.html]

Supplementary Data 

# One fledgling or two in the endangered Carnaby's Cockatoo (*Calyptorhynchus latirostris*): a strategy for survival or legacy from a bygone era?

## Supplementary Data

Supplementary Data

**Files in this Data Supplement:**

- Supplementary Data - Docx file
