# Supplementary material for: One fledgling or two in the endangered Carnaby's Cockatoo (Calyptorhynchus latirostris): a strategy for survival or legacy from a bygone era?
Source: Conserv Physiol. 2014 Feb 17;2(1):cou001. doi: 10.1093/conphys/cou001 (PMC4732493; doi:10.1093/conphys/cou001)
Supplement: Supplementary Data [file supp_cou001_cou001supp.docx]

Supplementary Table 1. The number of visits made each breeding season to each of seven study areas from 1969 to 2012. Carnaby’s Cockatoo nest hollows in the “Midlands” were in several localities and some were visited more often each year than others, hence a range of number of visits is given.

| Year | Coomallo | Manmanning | Tarwonga | Moornaming | Nereeno | “Midlands” | “Great Southern” |
| --- | --- | --- | --- | --- | --- | --- | --- |
| 1969 | 3 | 7 | 7 | 3 |  |  |  |
| 1970 | 13 | 11 | 10 | 7 |  |  |  |
| 1971 | 11 | 12 | 2 |  |  |  |  |
| 1972 | 25 | 20 |  |  |  |  |  |
| 1973 | 21 | 21 |  |  |  |  |  |
| 1974 | 21 | 24 |  |  |  |  |  |
| 1975 | 10 | 12 |  |  | 8 |  |  |
| 1976 | 11 | 9 |  |  | 11 |  |  |
| 1977 | 5 |  |  |  | 2 |  |  |
| 1978 | 1 |  |  |  | 2 |  |  |
| 1979 |  |  |  |  | 2 |  |  |
| 1980 |  |  |  |  | 2 |  |  |
| 1981 | 2 |  |  |  |  |  |  |
| 1982 | 2 |  |  |  |  |  |  |
| 1983 | 2 |  |  |  |  |  |  |
| 1984 | 2 |  |  |  |  |  |  |
| 1985 | 2 |  |  |  |  |  |  |
| 1986 | 2 |  |  |  |  |  |  |
| 1988 | 2 |  |  |  |  |  |  |
| 1989 | 2 |  |  |  |  |  |  |
| 1990 | 2 |  |  |  |  |  |  |
| 1994 | 2 |  |  |  |  |  |  |
| 1996 | 2 |  |  |  |  | 3-9 |  |
| 1997 |  |  |  |  |  | 3-9 |  |
| 1998 |  |  |  |  |  | 3-9 |  |
| 2003 | 1 |  |  |  |  | 2 |  |
| 2005 |  |  |  |  |  | 2 |  |
| 2006 |  |  |  |  |  | 2 |  |
| 2007 |  |  |  |  |  | 2 |  |
| 2008 |  |  |  |  |  | 2 | 1 |
| 2009 | 2 |  |  |  |  | 2 | 1 |
| 2010 | 2 |  |  |  |  | 2 | 1 |
| 2011 | 2 |  |  |  |  | 2 | 1 |
| 2012 | 2 |  |  |  |  | 2 | 2 |
